# A novel surgical approach for hypopharyngeal carcinoma resection via the paraglottic space

**DOI:** 10.1186/s12893-021-01223-1

**Published:** 2021-05-03

**Authors:** Wenming Li, Dongmin Wei, Ye Qian, Shengda Cao, Dayu Liu, Dapeng Lei, Xinliang Pan

**Affiliations:** Department of Otorhinolaryngology, Qilu Hospital of Shandong University, NHC Key Laboratory of Otorhinolaryngology (Shandong University), 107 Wenhua Xilu, Jinan, 250012 Shandong China

**Keywords:** Hypopharyngeal squamous cell carcinoma, Paraglottic space, Laryngeal preservation, Dysphagia, Pharyngocutaneous fistula

## Abstract

**Background:**

Conservative surgery has proven advantageous in controlling hypopharyngeal squamous cell carcinoma (HSCC) and preserving speech and swallowing function in carefully selected patients, typically with early T-stages diseases. A variety of modified surgical procedures or techniques have been proposed.

**Methods:**

In this study, we present a novel surgical approach for hypopharyngeal carcinoma resection utilizing the paraglottic space.

**Results:**

The paraglottic space approach can help expose neoplasms under direct vision and save mucosa during surgery while sufficiently preserving laryngeal function, thus benefiting postoperative swallowing and reducing complications. A large cohort of 426 patients with HSCC underwent surgical treatment at our institution using this approach, demonstrating an overall survival (OS) rate of 52.3% and low incidences of postoperative complications.

**Conclusions:**

This surgical approach can be applied in patients with the lesions that do not involve the paraglottic space.

**Supplementary Information:**

The online version contains supplementary material available at 10.1186/s12893-021-01223-1.

## Background

Hypopharyngeal squamous cell carcinoma (HSCC) is among the most malignant tumors in the head and neck and is generally associated with a poor prognosis, with a 5-year survival rate of approximately 30% to 35% [1]. The dismal outcome is largely credited to the absence of symptoms in the tumor’s early stage, as well as the tumor’s tendency for submucosal spread and early cervical lymph node metastasis [2].

The pyriform sinus is the area most vulnerable to HSCC, accounting for 70% of all cases, followed by the postcricoid region (15%–20%) and the posterior wall of the hypopharynx (10%–15%). The treatment for HSCC comprises mainly surgery and postoperative radiotherapy [3]. A variety of surgical procedures have been proposed for HSCC treatment, including total laryngopharyngectomy and conservation surgery, such as partial laryngopharyngectomy, and transoral laser surgery (TOLS) [4]. Conservative surgery has gained considerable attention from researchers and been extensively modified because it has proven effective in controlling this malignancy and preserving speech and swallowing function in carefully selected patients, typically those with early T-stages disease [5].

Lateral pharyngotomy has been widely used for HSCC resection since the 1950s because it can provide easy access to the pharyngeal cavity, supraglottic area, and oropharynx [6] to achieve adequate excision. However, entry into the hypopharynx via transection of the inferior constrictor muscle tends to sacrifice excessive pharyngeal mucosa, potentially increasing morbidity with severe postoperative complications [7], such as dysphagia and pharyngocutaneous fistula.

We have developed a novel approach for HSCC resection via the paraglottic space, which can help extirpate the tumor under direct vision in selected patients and avoid extensive excision of healthy mucosa. In this retrospective study, this novel approach was performed on a cohort of 426 patients in our institution between January 2005 and December 2015, with positive results for laryngeal preservation, complication control, and overall survival (OS).The application of this approach is best indicated when neoplasms don’t extend to the contralateral aryepiglottic fold, paraglottic space, or posterior pharyngeal wall.

## Methods

### Patients and data

Between January 2005 and December 2015, a total of 426 patients with HSCC were enrolled and received surgical treatment using the novel approach in the Department of Otolaryngology, Qilu Hospital of Shandong University, Jinan, China.

Patient and clinical data, such as sex, age, and TNM stage, were reviewed from medical records. The TNM stage was determined by two independent senior surgeons based on pathological examination in accordance with the American Joint Committee on Cancer Staging Manual (2019, 8th Edition). All the enrolled patients must have a definitive pathologic diagnosis of HSCC before surgery. And none of the included patients received chemotherapy or radiotherapy prior to surgery. Patients with severe intercurrent cadiocerebrovascular diseases or synchronous esophageal carcinoma were excluded from this study. Patients were fully informed and signed written consent for this research when admitted. This study was approved by the Institutional Review Board of Qilu Hospital of Shandong University (No.: 2018106).

### Description of the surgical procedure

After the induction of general anesthesia following tracheotomy, patients underwent ipsilateral selective neck dissection. Additionally, contralateral neck dissection was performed in those with suspicious lymph node involvement on the opposite side of the neck.

To achieve exposure of the ipsilateral laminae of the thyroid cartilage, the ipsilateral thyroid lobe was dissociated, followed by removal of the greater cornu of the hyoid bone and resection of the inferior pharyngeal constrictor along the posterior border of the thyroid cartilage. Next, the thyroid cartilage was longitudinally incised and divided into two parts, i.e.*,* the anterior 2/3 and the posterior 1/3. The paraglottic space was easily exposed by pulling outward the posterior part of the thyroid cartilage and excising the inside fat tissue along the lateral surface of the thyroarytenoid muscle. It was also important to note whether the paraglottic space was invaded. Once the paraglottic space was found involved, total laryngectomy with partial pharyngectomy was alternatively performed.

For carcinomas located mainly in the lateral wall of the pyriform sinus, retracting the thyroid cartilage can keep the ipsilateral pyriform sinus detached from the larynx. Thus, the selection of an approximate site in the paraglottic space to access the pharyngeal cavity allows tumor excision with adequate margins under direct vision. The excision usually includes the lateral and partial medial walls of the pyriform sinus and the split thyroid cartilage. For neoplasms confined only to the medial aspect of the pyriform sinus, it is necessary to sufficiently skeletonize the lateral surface of the thyroarytenoid until the emergence of the arytenoid so that the deep margin of the tumor can be completely resected. Subsequently, the tumor can be directly visualized and resected through either the incision behind the posterior border of the thyroid cartilage or through access to the pharyngeal cavity from the free edge of the aryepiglottic fold. The total pyriform sinus, paraglottic space, outer mucosa of the aryepiglottic fold, and detached thyroid cartilage are all involved in the resection.

Primary closure is performed after removal of the tumor to reconstruct the pharynx, and the ipsilateral uninvolved thyroid lobe is usually sutured to constitute a reinforcement plane. Alternatively, the pectoralis major myocutaneous flap can be used to repair oversized defects.

### Adjuvant radiotherapy

In China, the treatment of HSCC patients with T1, N0 lesions and no adverse features are generally recommended in accordance with National Comprehensive Cancer Network (NCCN) guidelines, while other patients are preferentially recommended for postoperative radiotherapy for 4–6 weeks. In this cohort, patients with T1, N0 lesions (n = 13) didn’t receive postoperative radiotherapy as advised. Radiotherapy was delivered to the remaining patients (n = 380), except for 33 patients who refused treatment. PGTVnx and PGTVnd received 66.00–73.92 Gy in 33 fractions (2.00–2.24 Gy/fraction). PTV1 received 60.06–66.0 Gy in 33 fractions (1.82–2.00 Gy/fraction) while PTV2 received 50 Gy in 28 fractions (1.82 Gy/fraction). No more than 5% of PTV volume received more than 110% of the prescribed dose. All patients were treated with one fraction daily for 5 days per week. Organs at risk included the spinal cord, parotid glands, brainstem, temporal lobes, temporomandibular joints, mandible, optimized on the basis of the dose-volume constraint criterion in the Radiation Therapy Oncology Group 0225 (RTOG0225) protocol.

### Follow-up

Patient follow-up data were collected when the patients periodically attended the clinic or by telephone until January 1, 2018. Overall survival (OS) was the key endpoint of interest in this study and was defined as the time (indicated in months) from the date of the operation to death from any cause or the last follow-up. Individuals who were alive at the end of the study or lost to follow-up were regarded as censored. Patients lost to follow-up accounted for 6.6% (28/426).

### Statistical analysis

Chi-square or Fisher’s exact tests were used to determine differences in clinical characteristics, including T classification and N classification, between patients with and without laryngeal preservation, and the impact of T stage on laryngeal function preservation. Survivals (overall survival: OS and disease-free survival: DFS) were analyzed using the Kaplan–Meier method with the log-rank test and Cox-regression hazard model. The SPSS software 19.0 (SPSS, Inc., Chicago, IL, USA) was used for all statistical analyses. P < 0.05 was defined as statistically significant.

## Results

**Fig. 1:**
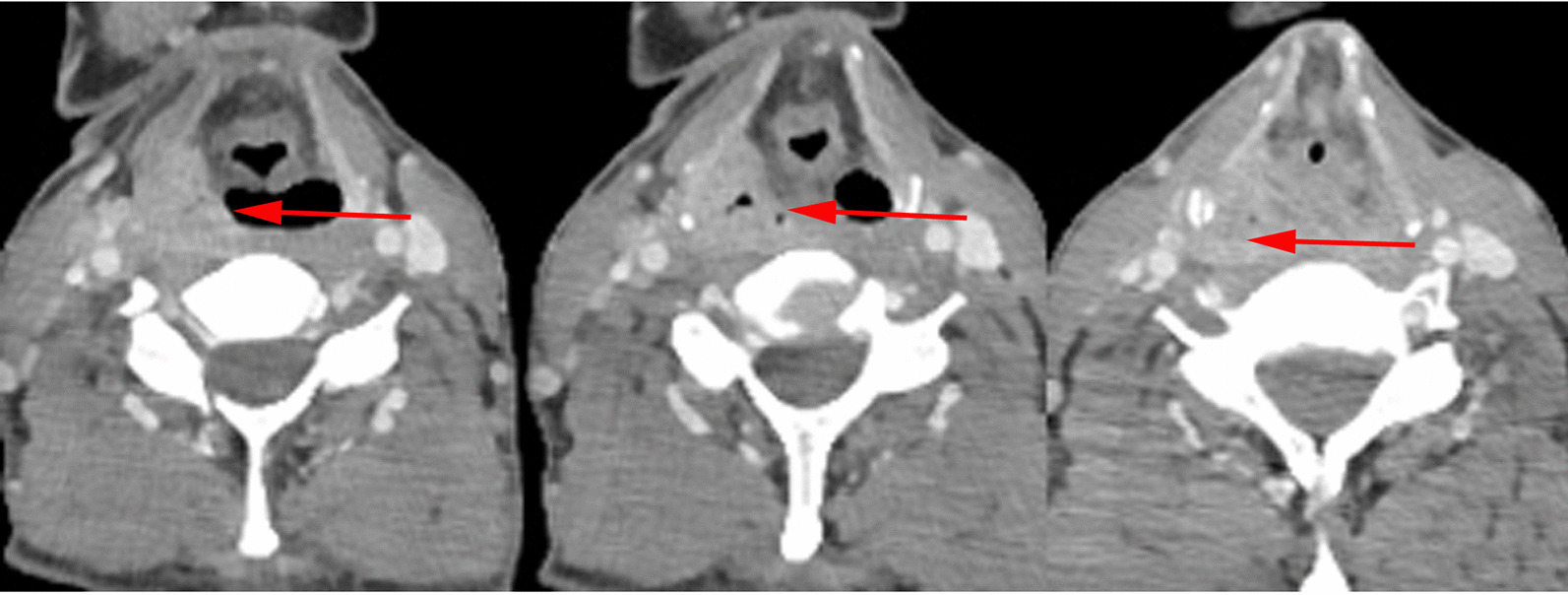
Preoperative contrast enhanced CT of one patient who was treated with the paraglottic approach. The arrow indicates the location of the tumor. The paraglottic space was identified not completely invaded and the esophagus was not involved

**Fig. 2:**
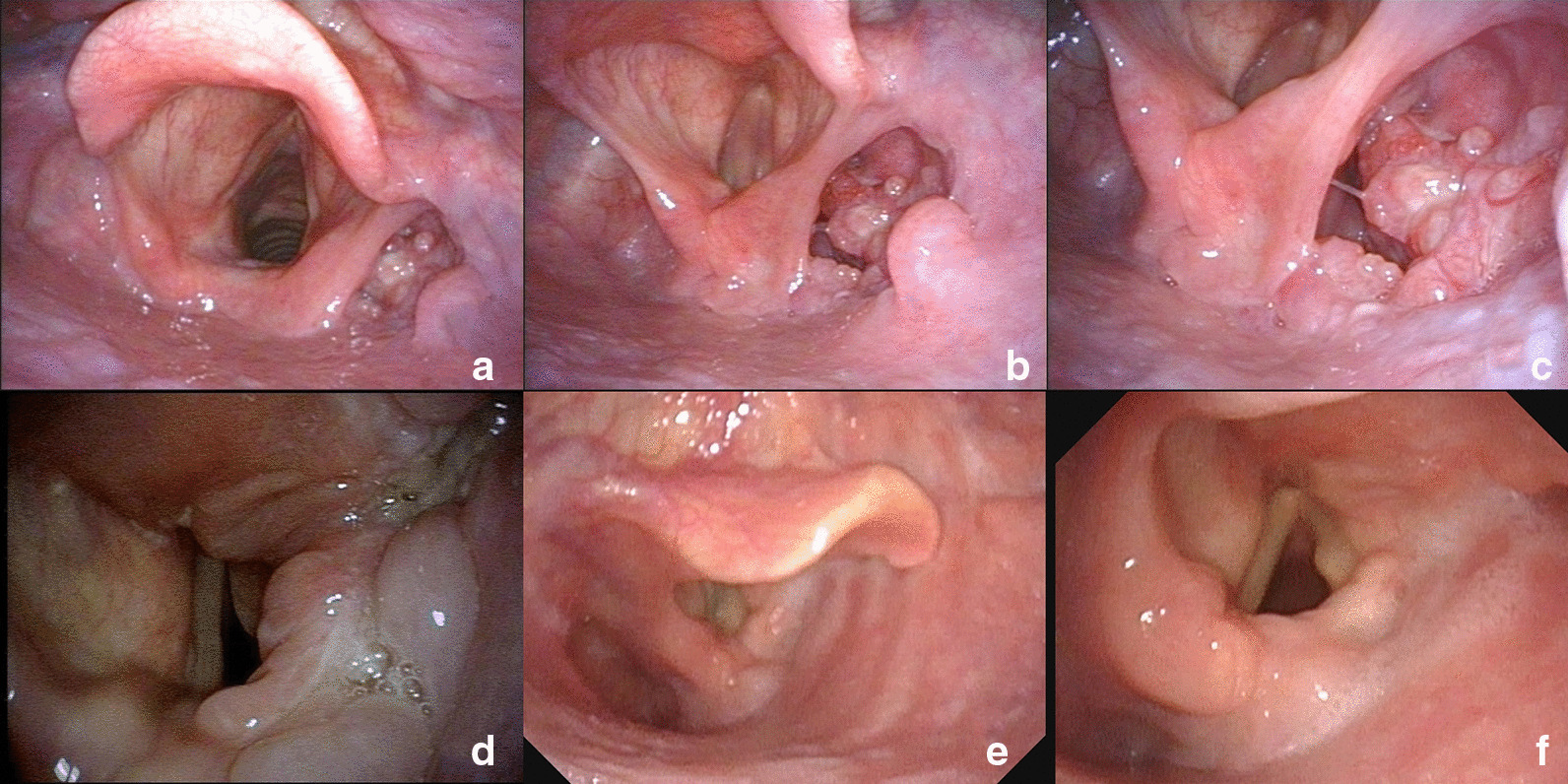
Representative pre- and postoperative laryngoscopic images of the same case. **a**, **b**, **c** The preoperative examination showed the tumor was confined to the right pyriform fossa without involving the laryngeal cavity. **d** The anastomotic stoma of the right pyriform fossa still appeared edematous 3 months after the operation. **e**, **f** The edema has completely disappeared and the mobile left vocal cord was observed 1 year after the operation

**Fig. 3:**
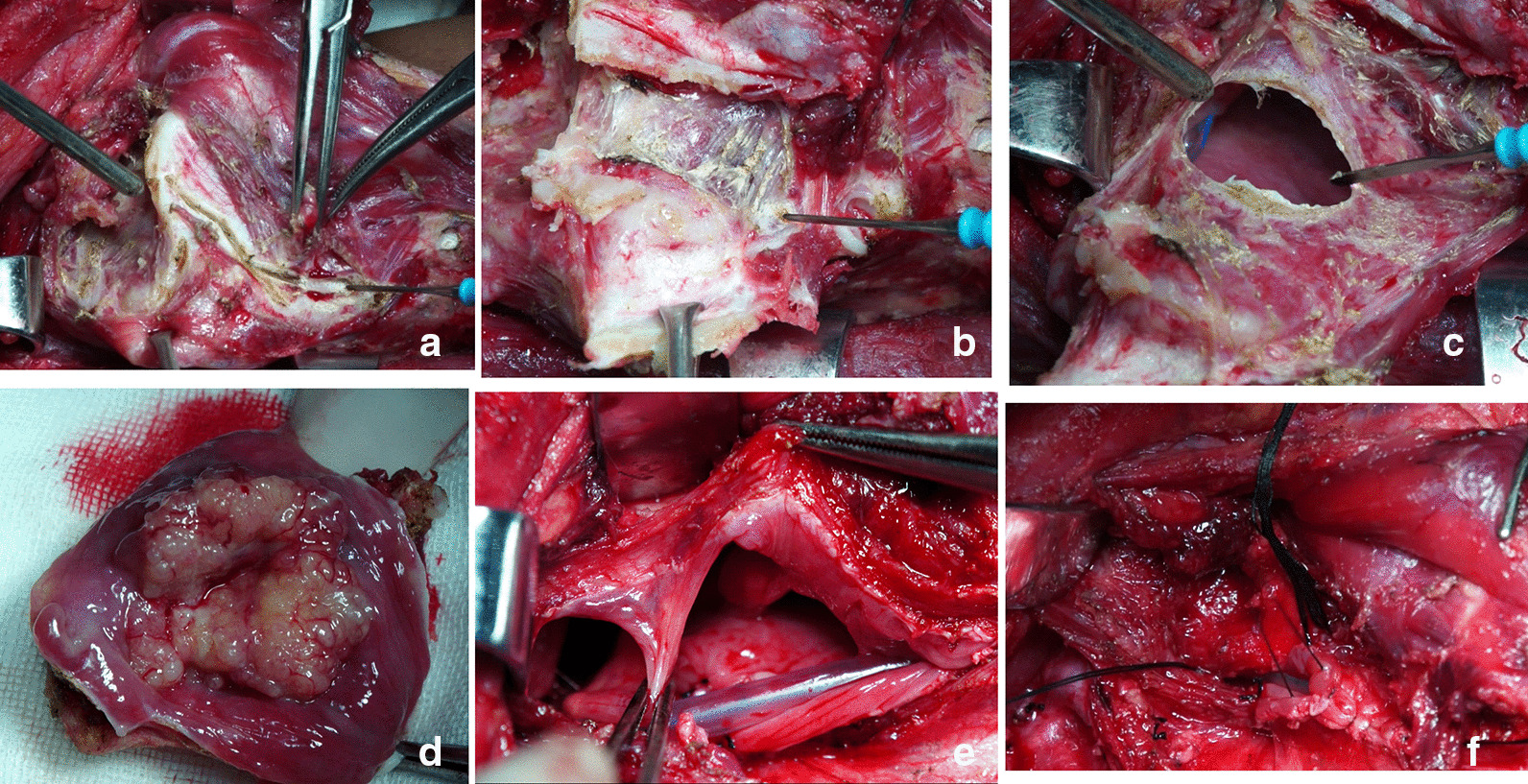
Key steps of the operation. **a** Exposure of the posterior margin of the thyroid cartilage plate. **b** Pulling the thyroid cartilage plate outward to expose the paraglottic space. **c** Separating from the paraglottic space and entering the pharyngeal cavity, resecting the tumor under direct vision. **d** Assessing the tumor margin. **e** The aryepiglottic fold is complete after tumor resection, and the pharyngeal cavity is widened by several stitches in the upper and lower mucosa of the posterior pharyngeal wall. **f** Closing the pharyngeal cavity with the pharyngeal mucosa suture

**Fig. 4:**
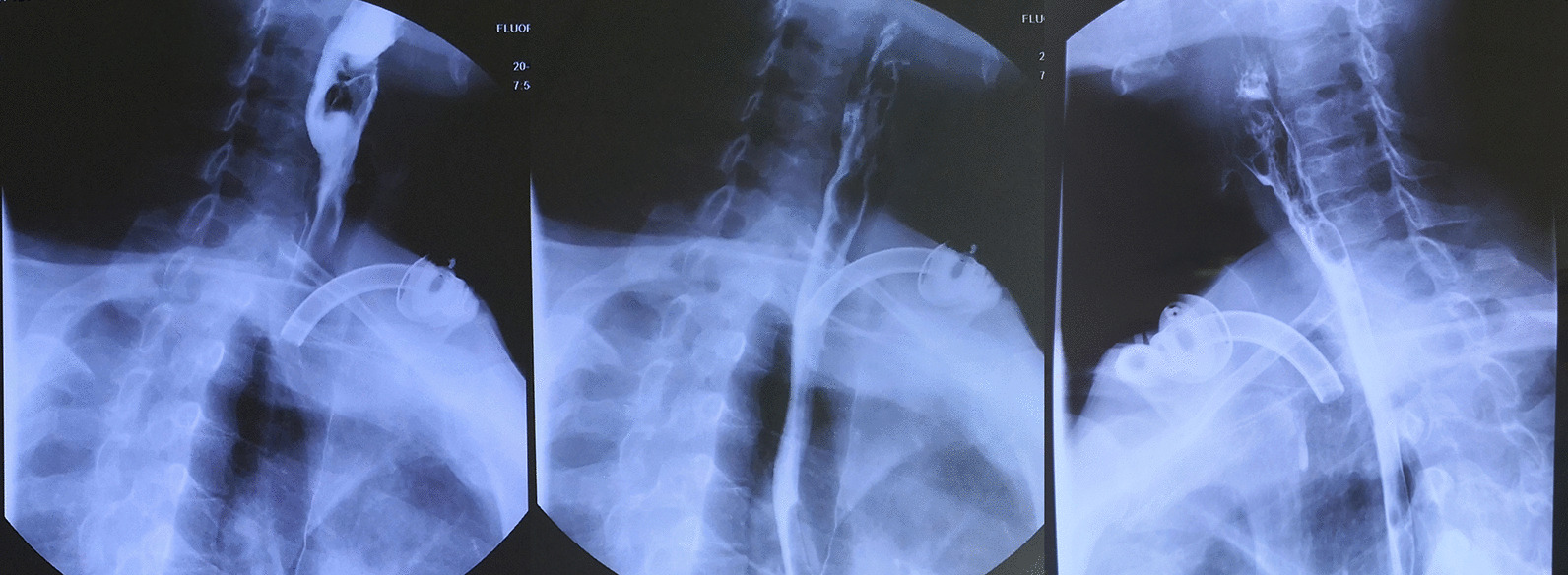
Barium swallow procedure with Ultravist 300; the contrast agent passed through the pharynx smoothly

The preoperative contrast enhanced CT of a representative individual who was enrolled in this study was shown in Fig. 1. Judging from his CT imaging, we observed that the tumor was located in the pyriform sinus (indicated as the arrow) without involvement of the ipsilateral paraglottic space or the esophagus. Moreover, the preoperative laryngoscopy examination demonstrated that the laryngeal cavity of this patient was not invaded, either (Fig. 2a–c). Thus, this patient received the surgical treatment using the paraglottic approach and critical surgical steps were shown in Fig. 3. Postoperative laryngoscopy examination revealed that anastomotic stoma of his patient healed normally and his laryngeal function was well preserved (Fig. 2d–f). And his swallowing function recovered well as indicated by the Barium swallow procedure with Ultravist 300 (Fig. 4).

A total of 426 patients with hypopharyngeal carcinoma were included in this study. There were 384 men and 42 women ranging in age from 36 to 80 years (mean, 61 years). All the patients were examined by preoperative fiber-optic laryngoscopy and diseases was diagnosed by pathologic biopsy before the operation. Among the cohort, the medial wall of the pyriform sinus was the most frequently affected site (205/426), followed by the lateral wall of the pyriform sinus (160/426), and 61 patients had involvement of both the lateral and medial walls of the pyriform sinus. The TNM staging based on postoperative pathological examination and other clinical characteristics of participants were summarized in Tables 1 and 2, respectively. None of the patients had distant metastasis (M = 0).

**Table 1:** TNM stage classification of the patients

N classification	No of patients by T classification				Total
	pT1	pT2	pT3	pT4	
pN0	13	36	22	13	84
pN1	29	65	13	9	116
pN2	6	92	87	32	217
pN3		7	1	1	9
Total	48	200	123	55	426

**Table 2:** Clinical characteristics of patients

Variable	No. of patients (%)
Sex	
Male	384
Female	42
Age, years	
< 55	90
55–65	222
> 65	114
Smoking history	
Ever	352
Never	74
Alcohol consumption	
> 50 g/d	363
< 50 g/d	63
Neck dissection	
Unilateral	238
Bilateral	188
Pathologic differentiation	
Well-differentiated	90
Moderately differentiated	192
Poorly differentiated	144
Cervical metastasis	
Yes	342
No	84

Laryngeal preservation was achieved in 346 patients, accounting for 81.2%.The postoperative pathologic analysis for all patients demonstrated squamous cell carcinoma, including 90 patients with well-differentiated tumors, 192 patients with moderately differentiated tumors, and 144 patients with poorly differentiated tumors. Cervical lymph node metastasis was identified in 80.2% of the cohort. No deaths occurred during the perioperative period. The effect of T stage on laryngeal preservation rate was further explored, and our results showed that T stage significantly affected laryngeal preservation rate (Table 3). As we expected, the patients with advanced T stage apparently had significantly impaired laryngeal function preservation, indicating that advanced T stage was an independent risk factor for disease recurrence (HR, 1.604, 95% CI 1.029–2.501, p = 0.037) (Additional file 1: Table S1).

**Table 3:** The influence of T stage on the rate of laryngeal function preservation

T stage	Laryngeal function preservation		χ^2^	P value
	Yes (No., %)	No (No., %)		
T1	48 (100%)	0 (0%)	45.68	< 0.001
T2	178 (89.5%)	21 (10.5%)		
T3	82 (66.7%)	41 (33.3%)		
T4	36 (65.5%)	19 (34.5%)		

**Fig. 5:**
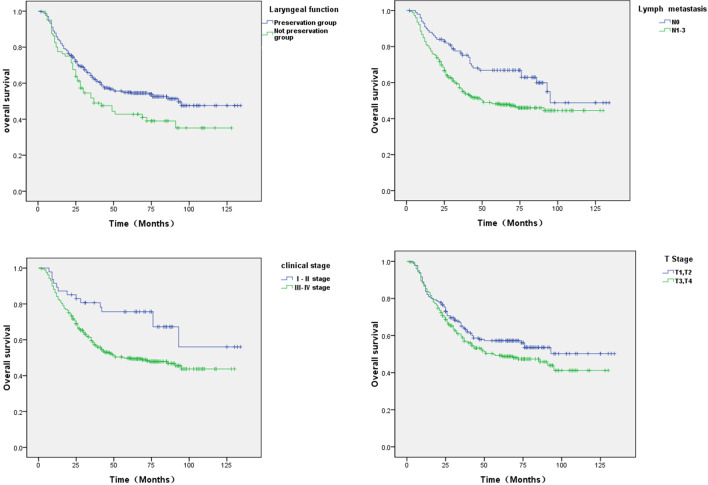
Kaplan–Meier estimates for survival of the included patients

The main postoperative complications in the study included cervical subcutaneous infection (n = 13), hemorrhage in the tracheotomy region (n = 4), and pharyngocutaneous fistula (n = 29) (Table 4). In the follow-up period, the OS rate reached 52.3%:175 patients died, 223 remained alive, and 28 were lost to follow-up. The OS rate for the group of patients with laryngeal preservation was significantly higher than that for the group without laryngeal preservation (54.9% vs. 41.3%, P = 0.034). For the patients with laryngeal preservation surgery, the 5-year OS was 54.4%, and the 3-year OS was 61.7%. The 5-year and 3-year DFS of those patients were 53.5% and 56.6%, respectively. Thus, the laryngectomy-free pharyngotomy using the paraglottic approach might indicate a clinical benefit in survival and locoregional control of patients with HSCC. We also analyzed the relationship between clinical characteristics and OS and found that both clinical stage (P = 0.01) and lymph node metastasis (P = 0.004) showed a negative correlation with patient prognosis (Fig. 5).

**Table 4:** Patient outcomes

Variable	No. of patients (%)
Pharyngocutaneous fistula	29 (6.8)
Postoperative infection	13 (3.1)
Chyle leakage	9 (2.1)
Hemorrhage in the tracheotomy region	4 (0.9)
Granulation hyperplasia of the pharynx	4 (0.9)
Preserved laryngeal function	346 (81.2)
Decannulation	272/346 (78.6)
Restore to oral diet (d)	
FOSS* score	16.4
0–2	365 (85.7)
3–4	45 (10.6)
5	16 (3.7)

FOSS*, functional outcomes wallowing scale [8]

Of the 346 patients with laryngeal preservation, 268 were extubated within 12 months after the operation. The tracheal cannulas of only 4 patients were removed approximately 24 months after the operation. Unfortunately, dyspnea occurred in 6 patients within 2 weeks of extubation. Patients were advised to begin oral intake 2 to 3 weeks after surgery. Only 16 patients developed dysphagia, among whom locoregional recurrence occurred in 12 and granulation tissue hyperplasia occurred in 4. A total of 45 patients had to maintain a liquid diet or coughed when eating. The recovery rate of swallowing function was 85.6% (Table 3).

## Discussion

HSCC is a highly malignant carcinoma of the head and neck region because of its location and potential for metastatic spread, among which pyriform sinus carcinoma represents 70%. The pyriform sinus is anatomically adjacent to the larynx and is responsible for swallowing, pronunciation, breathing, and other functions. It is well established that partial or even total preservation of the larynx has no negative effect on the lifespan of carefully selected HSCC patients [8]. It remains an important issue to remove HSCC while preserving swallowing and laryngeal functions and decreasing the risk of complications [9]. Here, we presented a new approach for HSCC resection via the paraglottic space on the basis of lateral pharyngotomy, aiming to provide a wide surgical field and achieve precise HSCC excision without damaging the larynx, as described in the Methods section.

The paraglottic space is seldom invaded in the context of T1-T2 HSCC lesions, making absence of such invasion the best indication for this surgery. Additionally, the paraglottic space approach can be applied in patients with T3-T4 lesions if the paraglottic space is not involved because the immobility of the ipsilateral vocal cord is attributed to pressure exerted by the bulk of the neoplasm, not tumor extension to the paraglottic space [2]. Enhanced computed tomography (CT) is an invaluable method for detecting whether the paraglottic space or thyroid cartilage is invaded. For neoplasms arising from the pyriform sinus or the aryepiglottic fold, the supraglottic hemilaryngopharygectomy (SCHLP) is most often performed. Several research reported similar results with 5-year OS rates about 50%. Laccourreye et al. reported that a cohort of patients with selected T2 lesions of pyriform sinus carcinoma were treated with the SCHLP, with a 5-year disease-specific survival rate of 56% [10]. Likewise, in another study, 48 cases with T1 or T2 staging of pyriform sinus carcinoma underwent the same surgery following induction chemotherapy, and the 5-year OS rate was reported 47% by Chevalier D and colleagues [11]. In comparison to these studies, the OS rate reached 52.3% in our study covering all T stages of patients, and the 5- or 3-year OS and DFS were satisfactory, indicating that this novel approach has a proven benefit in controlling this malignancy.

For neoplasms confined to the lateral wall of the pyriform sinus, the larynx is seldom involved. Entrance into the pharyngeal cavity using the paraglottic approach can expose lesions from the ventral perspective, which provides a wide surgical field. Tumors originating from the medial wall of the pyriform sinus tend to invade the larynx, especially the paraglottic space and aryepiglottic fold. The paraglottic approach can be used to completely excise the deep margin of the tumor while entering the pharynx. The tumor can be kept away from the larynx along with the detached thyroid cartilage by retraction, also aiding surgeons in complete tumor resection under direct vision. Standard lateral pharyngotomy usually exposes the lesion from the dorsal view, and the access site may not be appropriate for tumor excision, sacrificing too much normal mucosa or being too close to the lesion. It is difficult for surgeons to observe the actual involvement of the tumor from the dorsal perspective. If the paraglottic space is observed to be invaded during the operation, it is essential to terminate this approach, and standard lateral pharyngotomy can alternatively be used. Partial laryngopharyngectomy can also be applied in patients in which the larynx is affected. In addition, when the defect of the pharynx is too large to be repaired with primary sutures, the pectoralis major myocutaneous flap is our preferred choice for reconstruction of the pharynx.

Historically, a variety of conservative surgical procedures with different characteristics have been proposed. However, nearly all of these procedures involve hemilaryngectomy, which can yield significant side effects, such as hoarseness and aspiration. For example, Iwai and colleagues performed a prospective study involving seven patients, in which patients were treated with resection of the affected side of the hypopharynx as well as the adjacent supraglottic and glottic larynx, even the superior 2/3 of the cricoid [12]. Later, Hirano et al. [13] and Yoo et al. [14] separately proposed partial laryngopharyngectomy for pyriform sinus cancer for larynx preservation. Additionally, Laccourreyeet al reported supracricoid hemilaryngopharyngectomy in 34 patients with T2 disease, which was quite helpful for voice preservation [15]. Compared with these procedures, the paraglottic space approach allows preservation of the cricoid, vocal cord, and cricoarytenoid joint, thus achieving more than functional laryngeal preservation, which is greatly favorable for postoperative quality of voice and reduction of aspiration problems.

In our study, application of the paraglottic approach significantly reduced one of the serious postoperative complications, i.e.*,* pharyngocutaneous fistula. In our opinion, after adequate excision, the paraglottic approach can help save more normal mucosa, as discussed above, which ensures a larger space in the new pharyngeal cavity and contributes greatly to lowering the tension in the sutures. The lower incidence of pharyngocutaneous fistula can also be partly attributed to the thyroid lobe, which is used as a reinforcement plane for the new pharynx during the operation. Moreover, our functional outcome swallowing scale (FOSS) results showed that the swallowing function in patients receiving this approach was well maintained, which we believe was mainly ascribed to the large cavity of the new pharynx.

In conclusion, as a novel technique for the conservative treatment for HSCC, the paraglottic approach has a proven benefit in controlling HSCC and decreasing the likelihood of severe postoperative complications. It also contributes to the recovery of the swallowing function after surgery, which is of significance to quality of life. Moreover, a much larger cohort was involved in our study than in previous studies, making our conclusion more convincing. This approach can be proposed as a promising candidate for resection of early T stages of HSCC.

## Supplementary Information

**Additional file 1: Table S1.** The impact of clinical characteristics on 5-year DFS.

## Data Availability

The data that support the findings of this study are available from the corresponding author upon reasonable request.

## Abbreviations

OS: Overall survival
HSCC: Hypopharyngeal squamous cell carcinoma
CT: Computed tomography
FOSS: Functional outcome swallowing scale

## References

[1] Garneau JC, Bakst RL, Miles BA (2018). Hypopharyngeal cancer: a state of the art review. Oral Oncol.

[2] Segas JV, Hantzakos AG, Tzagaroulakis AM, Adamapoulos GK (2001). A novel technique in the operative treatment of pyriform sinus carcinoma. Acta Otolaryngol.

[3] Takes RP, Strojan P, Silver CE (2012). Current trends in initial management of hypopharyngeal cancer: the declining use of open surgery. Head Neck.

[4] Lefebvre JL (2009). Surgery for Laryngopharyngeal SCC in the era of organ preservation. Clin Exp Otorhinolaryngol.

[5] Lefebvre JL (2000). What is the role of primary surgery in the treatment of laryngeal and hypopharyngeal cancer? Hayes Martin Lecture. ArchOtolaryngol Head Neck Surg.

[6] Laccourreye O, Villeneuve A, Rubin F, Holsinger FC (2019). Lateral pharyngotomy. Eur Ann Otorhinolaryngol Head Neck Dis.

[7] Kedous S, Turki S, Elhedhili F (2019). Risk factor of pharyngocutaneous fistula. Tunis Med.

[8] Freeman RB, Marks JE, Ogura JH (1979). Voice preservation in treatment of carcinoma of the pyriform sinus. Laryngoscope.

[9] Triboulet JP, Mariette C, Chevalier D, Amrouni H (2001). Surgical management of carcinoma of the hypopharynx and cervical esophagus: analysis of 209 cases. Arch Surg.

[10] Laccourreye O, Me’rite-Drancy A, Brasnu D (1993). Supracricoidhemilaryngopharyngectomy in selected pyriform sinus carcinoma staged as T2. Laryngoscope.

[11] Chevalier D, Watelet JB, Darras JA, Piquet JJ (1997). Supraglottic hemilaryngopharyngectomy plus radiation for the treatment of early lateral margin and pyriform sinus carcinoma. Head Neck.

[12] Iwai H, Koike Y, Nagahara K (1975). Subtotal pharyngolaryngectomy conservation surgery for carcinoma of sinus pyriformis extending toward the larynx. Arch Otorhinolaryngol.

[13] Hirano M, Kurita S, Yoshida T, Tanaka H, Tai Y (1988). Partial laryngopharyngectomy for piriform sinus carcinoma. Technique and preliminary results. Auris Nasus Larynx.

[14] Yoo SJ, Lee SH, Koh KS, Kim SY (2000). Larynx preservation surgery in pyriform sinus cancer. IntSurg.

[15] Laccourreye O, Merite-Drancy A, Brasnu D, Chabardes E, Cauchois R, Menard M (1993). LaccourreyeH. Supracricoidhemilaryngopharyngectomy in selected pyriform sinus carcinoma staged as T2. Laryngoscope.

