# Supplementary material for: A novel surgical approach for hypopharyngeal carcinoma resection via the paraglottic space
Source: BMC Surg. 2021 May 3;21:230. doi: 10.1186/s12893-021-01223-1 (PMC8094522; doi:10.1186/s12893-021-01223-1)
Supplement: Supplementary file 1 — Additional file 1: Table S1. The impact of clinical characteristics on 5-year DFS. [file 12893_2021_1223_MOESM1_ESM.docx]

| T stage | P | HR | 95% CI |
| --- | --- | --- | --- |
| T1(Ref.) |  | 1.000 |  |
| T2 | 0.300 | 0.313 | 0.146-1.671 |
| T3 | 0.993 | 1.002 | 0.649-1.547 |
| T4 | 0.037 | 1.604 | 1.029-2.501 |

Additional file 1: Table S1. The impact of clinical characteristics on 5-year

DFS.
